# Clinical Evaluation of the Inverse Planning System Utilized in Gamma Knife Lightning

**DOI:** 10.3389/fonc.2022.832656

**Published:** 2022-02-23

**Authors:** Taoran Cui, Ke Nie, Jiahua Zhu, Shabbar Danish, Joseph Weiner, Anupama Chundury, Nisha Ohri, Yin Zhang, Irina Vergalasova, Ning Yue, Xiao Wang

**Affiliations:** ^1^ Department of Radiation Oncology, Rutgers-Cancer Institute of New Jersey, Rutgers-Robert Wood Johnson Medical School, New Brunswick, NJ, United States; ^2^ Department of Radiation Oncology, Reading Hospital, Tower Health, West Reading, PA, United States; ^3^ Department of Neurosurgery, Jersey Shore University Medical Center, Neptune City, NJ, United States

**Keywords:** inverse planning, Gamma Knife Icon™ ^®^, GammaPlan^®^, Gamma Knife^®^ Lightning, stereotactic radiosurgery (SRS), pituitary adenoma, vestibular schwannoma, brain metastases (BM)

## Abstract

**Objectives:**

The purpose of this study is to independently compare the performance of the inverse planning algorithm utilized in Gamma Knife (GK) Lightning Treatment Planning System (TPS) to manual forward planning, between experienced and inexperienced users, for different types of targets.

**Materials and Methods:**

Forty patients treated with GK stereotactic radiosurgery (SRS) for pituitary adenoma (PA), vestibular schwannoma (VS), post-operative brain metastases (pBM), and intact brain metastases (iBM) were randomly selected, ten for each site. Three inversely optimized plans were generated for each case by two experienced planners (OptExp1 and OptExp2) and a novice planner (OptNov) using GK Lightning TPS. For each treatment site, the Gradient Index (GI), the Paddick Conformity Index (PCI), the prescription percentage, the scaled beam-on time (sBOT), the number of shots used, and dosimetric metrics to OARs were compared first between the inversely optimized plans and the manually generated clinical plans, and then among the inversely optimized plans. Statistical analyses were performed using the Student’s t-test and the ANOVA followed by the post-hoc Tukey tests.

**Results:**

The GI for the inversely optimized plans significantly outperformed the clinical plans for all sites. PCIs were similar between the inversely optimized and clinical plans for PA and VS, but were significantly improved in the inversely optimized plans for iBM and pBM. There were no significant differences in the sBOT between the inversely optimized and clinical plans, except for the PA cases. No significant differences were observed in dosimetric metrics, except for lower brain V_12Gy_ and PTV D_98%_ in the inversely optimized plans for iBM. There were no noticeable differences in plan qualities among the inversely optimized plans created by the novice and experienced planners.

**Conclusion:**

Inverse planning in GK Lightning TPS produces GK SRS plans at least equivalent in plan quality and similar in sBOT compared to manual forward planning in this independent validation study. The automatic workflow of inversed planning ensures a consistent plan quality regardless of a planner’s experience.

## Introduction

The Leksell Gamma Knife (GK) is considered an effective stereotactic radiosurgery (SRS) platform for various cranial diseases. Although initially designed for small and well-circumscribed lesions, such as targets in functional brain surgery or solitary brain metastasis (1), GK continues to evolve with advanced technologies of variable collimator sizes, motor-driven sector, and image-guided cone-beam CT (CBCT), to allow for SRS for targets of non-spherical and irregular shapes. There have been excellent clinical outcomes reported on the management of post-operative brain metastases (2), acoustic neuroma (3), pituitary adenoma (4), and arteriovenous malformations (5) using GK SRS.

During an SRS treatment delivered by a GK Perfexion/ICON™ unit, 8 sectors with ^60^Co sources can be independently modulated with collimators of three different sizes to achieve a variable irradiated volume at the isocenter. A patient receiving GK SRS is supported and moved by a high-precision treatment couch which aligns the irradiation focal point to a pre-defined cranial location, and the radiation is delivered at the location with a given set of collimators, which is usually referred as a “shot”. A single shot or multiple shots with various weights can be used to achieve desired coverage or conformity for targets of different volumes or shapes. The treatment planning for GK SRS is therefore defined as the adjustments of collimator selection, isocenter location, and relative weight of multiple shots to create a GK SRS plan that meets the pre-defined requirements.

Traditionally, the design of a GK SRS plan is performed manually, in a forward, trial-and-error approach. The quality of a GK SRS plan generated in this approach significantly relies on the prior experiences of a planner, the time and effort spent on planning, and different planning approaches applied among various planners. Hence, the plan quality of a GK SRS plan usually suffers from inter-planner variabilities and it is often challenging to ensure consistent plan quality. In addition, the improvement of plan quality of GK SRS plans is usually accompanied by the inflation of beam-on time (BOT) (6) which could result in prolonged treatment time and patient discomfort. Therefore, it is desirable to have an efficient and effective automatic treatment planning system available for clinical application independent of planner’s experiences to assure consistent plan quality for GK SRS.

Multiple inverse planning approaches have been proposed for GK SRS using morphology guided (7), non-linear programming (8), multiresolution-level (9) techniques. Whereas these approaches presented promising results, they were never integrated with the treatment planning system (TPS) thus not ideal for real-time clinical application. More recently, a new inverse planning technology has become clinically available in the latest GK Lightning TPS which enables an automatic workflow for GK treatment planning with minimum manual inputs (10). While the initial study performed by the vendor has demonstrated encouraging results, only two independent studies on GK Lightning have been recently published (11, 12), and independent evaluations of the inverse planning technology with broader clinical relevance are still in need. In the current manuscript, we aim to evaluate this new inverse planning technology by comparing the inversely optimized plan generated with this technology to clinically approved forward-based plans for a variety of disease types. The inversely optimized plans generated by different planners with various experience levels were also compared for all plans to assess the readiness of this new technology.

## Materials and Methods

Patients treated with GK SRS at our institution for pituitary adenoma (PA), vestibular schwannoma (VS), postoperative surgical bed of brain metastases (pBM), and intact brain metastases (iBM) were randomly selected for this retrospective, institutional IRB approved (Pro2018000227) study.

Per departmental protocol, series of T1- and T2-weighted MRI were acquired prior to treatment planning. Gross tumor volumes (GTVs) were contoured as radiographic enhancements on contrast-enhanced T1-weighted FSPGR MRI in 1.5mm axial cuts, and verified on an independent series of contrast-enhanced T1-weighted MRI in coronal cuts. Clinical target volumes (CTVs) were the same as GTVs, except for post-operative brain metastasis cases, where there was no GTV and a CTV was created by adding a 2-mm isotropic margin to a surgical cavity. All the selected clinical plans were initially treated with frame fixation and therefore setup margin was not used. Therefore, planning target volumes (PTVs) was identical to CTVs. The original clinical plans were manually designed to achieve at least 99% target coverage by prescribed doses, unless at the discretion of physicians. The dose constraints to organs-at-risk (OARs) were strictly followed with AAPM Task Group report 101 (13), that D_0.035cc_<10 Gy and D_0.2 cc_<8 Gy for the optical apparatus, D_0.035cc_<15 Gy and D_0.5cc_<10 Gy for the brainstem, and D_0.035cc_<9 Gy for the cochlea.

To compare the differences between the manual forward planning and the new inverse planning technology, each patient was replanned by two experienced planners (OptExp1 and OptExp2) and one novice planner (OptNov) utilizing the inverse planning technology in the GK Lightning TPS (10). The experienced users have at least 5 years of GK planning experience, while the novice planner has less than 3 months experience. The implementation of the inverse planning technology was discussed in detail by Sjölund et al. (10). Briefly, a cost function is first constructed to include various planning objectives defined by a planner, including the prescription dose coverage of multiple targets, the maximum dose delivered to targets and OARs, low dose spillage, and BOT, in order to encourage higher target coverage and lower selectivity and to penalize higher dose to the OARs and longer BOT. The optimizer then adjusts the position and weight of each shot by minimizing the cost function using linear programming until an optimal solution to the cost function is achieved. Neither shot placement nor shot opening selection is required prior to optimization. Furthermore, multiple targets could be inversely optimized at the same time.

In this study, the same original clinical plan prescription dose was chosen to cover the target, and target coverage was selected as a mandatory constraint. Similarly, the maximum dose to OARs was defined using the same dose constraints as the approved clinical plan. There were no new requirements on the weights of low dose spillage or BOT, for both the weights were frequently adjusted by a planner in a trial-and-error approach. Manual modifications of shot position and weight after the optimization were allowed to either increase target coverage or reduce OAR dose, if necessary. The planning goal was to achieve at least 99% target coverage and as good a Paddick Conformity Index (PCI) (14) and Gradient Index (GI) (15) as possible, with the goal of PCI > 0.75 and GI < 3. In order to compare the BOT between the clinical and inversely optimized plans generated at different dose-rates, the BOTs were scaled to the same dose rate of 3.5 Gy per minute (sBOT).

The inversely optimized plans were compared to the original clinical plan using several metrics including target coverage, prescription percentage, PCI, GI, sBOTs, number of shots, and number of different utilized sectors. The prescription percentage is defined as the percentage of the dose maximum that is normalized with the prescription dose. D_0.035cc_ and D_0.5cc_ of the brainstem, D_0.035cc_ of the optic chiasm/nerves, and the ipsilateral cochlea were evaluated if these OARs were present. Furthermore, V_12Gy_ of the brain was also included for analysis for postoperative and intact brain metastases cases.

For each disease site, the Student’s t-test was first applied to compare the metrics between clinical and inversely optimized plans. Specifically, the Chi-square test was used to demonstrate if there was any difference in the numbers of sectors used per plan. Then, the one-way ANOVA test was performed to determine if there were any differences in the metrics among the inversely optimized plans created by different planners, followed by the post-hoc Tukey Test to determine the metrics of which specific inversely optimized plans were different. A p-value less than 0.05 was considered statistically significant.

## Results

### Dosimetric Metrics

**Figure 1:**
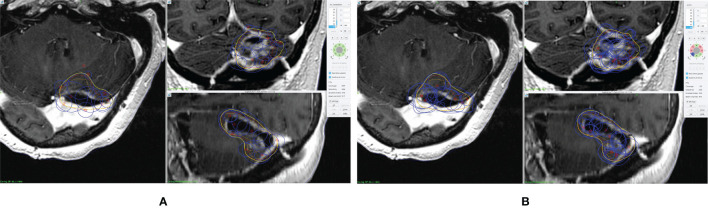
An example of prescription dose coverage and shot placement in **(A)** clinical plan and **(B)** inversely optimized plan. The surgical cavity is delineated in orange, and the CTV is in red. The blue lines represent the shots, and the yellow lines represent the isodose lines of the prescription dose.

Forty patients treated with GK SRS were identified from our institutional database with 10 patients for each of the pre-defined disease sites. The prior clinical plans were all manually generated without using any optimization algorithms. All optimized plans generated using the inverse planning technology of the GK Lighting TPS were reviewed to ensure that target coverage and dosimetric OAR constraints all met the clinical criteria. Although manual modification was allowed, none of the planners reported necessity of manual tweaking of shots, since all dosimetric criteria were met after iterative inverse optimization. A comparison of dose distribution and shot placement between the clinical plan and inversely optimized plan for a representative pBM case is shown in **Figure 1**. Compared to the clinical plan, the inversely optimized plan has similar coverage, but greater number of shots with different isocenters.

### Results for Each Site

**Figure 2:**
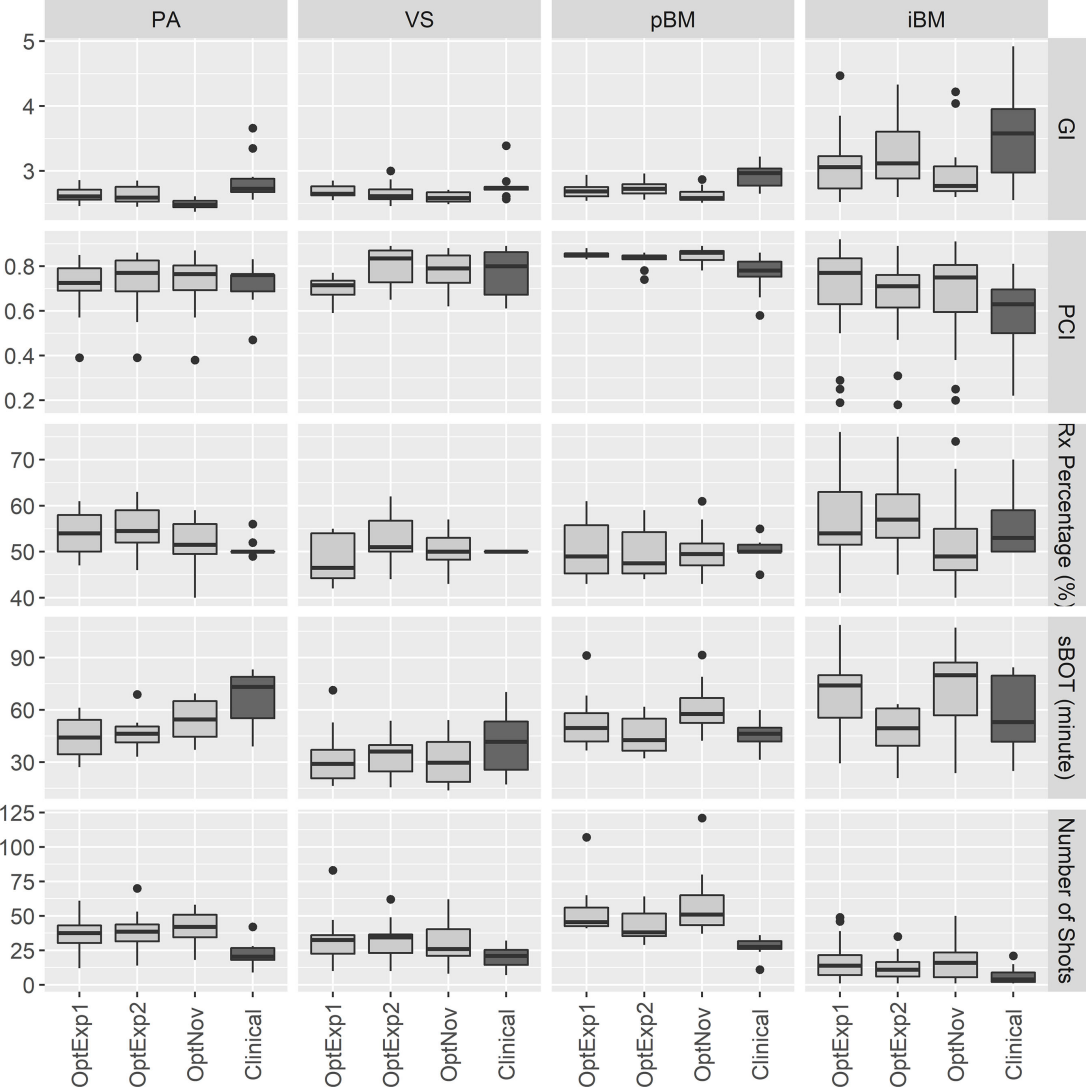
Boxplots of the plan qualities among the inversely optimized and clinical plans for all sites. PA, pituitary adenoma; VS, vestibular schwannoma; pBM, post-operative brain metastases; iBM, intact brain metastases; GI, gradient index; PCI, Paddick Conformity Index; RxPercentage, prescription percentage; sBOT, scaled beam-on time.

**Figure 2**, **Tables 1** and **2** summarized the comparison of plan qualities among the inversely optimized and clinical plans for patients treated for PA, VS, pBM, and iBM.

**Table 1:** Summary of the target metrics among the inversely optimized and clinical plans for all sites.

Metrics	Site	OptExp1	OptExp2	OptNov	Clinical	p-value Student’s t-test	ANOVA
GI	PA	2.64 ± 0.13 (2.46-2.86)	2.63 ± 0.14 (2.45-2.85)	2.49 ± 0.07 (2.37-2.61)	2.88 ± 0.35 (2.56-3.66)	**p < 0.05**	**p<0.05; OptNov**
	VS	2.68 ± 0.10 (2.55-2.85)	2.67 ± 0.16 (2.46-3.00)	2.59 ± 0.08 (2.49-2.71)	2.78 ± 0.23 (2.57-3.39)	**p < 0.05**	
	pBM	2.69 ± 0.13 (2.54-2.94)	2.73 ± 0.12 (2.56-2.96)	2.63 ± 0.12 (2.51-2.87)	2.93 ± 0.20 (2.65-3.22)	**p < 0.05**	
	iBM	3.13 ± 0.51 (2.52-4.47)	3.25 ± 0.54 (2.60-4.33)	2.95 ± 0.43 (2.60-4.22)	3.55 ± 0.68 (2.55-4.92)	**p < 0.05**	
PCI	PA	0.71 ± 0.14 (0.39-0.85)	0.72 ± 0.15 (0.39-0.86)	0.72 ± 0.15 (0.38-0.87)	0.72 ± 0.11 (0.47-0.83)		
	VS	0.70 ± 0.06 (0.59-0.77)	0.80 ± 0.09 (0.65-0.89)	0.78 ± 0.09 (0.62-0.88)	0.77 ± 0.11 (0.61-0.89)		**p<0.05; OptExp1**
	pBM	0.85 ± 0.01 (0.83-0.88)	0.83 ± 0.04 (0.74-0.86)	0.85 ± 0.03 (0.78-0.89)	0.77 ± 0.09 (0.58-0.86)	**p < 0.05**	
	iBM	0.71 ± 0.19 (0.19-0.92)	0.67 ± 0.16 (0.18-0.89)	0.68 ± 0.19 (0.20-0.91)	0.59 ± 0.15 (0.22-0.81)	**p < 0.05**	
RxPercentage (%)	PA	54.20 ± 5.05 (47.00-61.00)	55.10 ± 5.63 (46.00-63.00)	51.90 ± 5.53 (40.00-59.00)	50.70 ± 2.00 (49.00-56.00)		
	VS	48.30 ± 5.23 (42.00-55.00)	52.80 ± 5.81 (44.00-62.00)	50.40 ± 4.77 (43.00-57.00)	50.00 ± 0.00 (50.00-50.00)		
	pBM	50.50 ± 6.33 (43.00-61.00)	49.60 ± 5.87 (44.00-59.00)	50.20 ± 5.45 (43.00-61.00)	50.40 ± 2.50 (45.00-55.00)		
	iBM	56.77 ± 8.21 (41.00-76.00)	58.23 ± 7.27 (45.00-75.00)	51.58 ± 8.17 (40.00-74.00)	55.42 ± 6.11 (50.00-70.00)		**p<0.05; OptNov**
sBOT (min)	PA	44.12 ± 11.67 (27.06-61.17)	46.60 ± 9.98 (33.09-68.80)	54.68 ± 11.64 (37.07-69.42)	67.09 ± 15.53 (38.97-83.10)	**p < 0.05**	
	VS	33.22 ± 17.30 (16.36-71.30)	33.22 ± 11.73 (15.48-53.67)	31.15 ± 13.91 (13.71-54.13)	40.05 ± 17.65 (17.17-70.15)		
	pBM	53.25 ± 16.46 (36.69-91.17)	45.03 ± 10.96 (32.10-61.73)	61.49 ± 14.96 (42.24-91.49)	45.65 ± 7.79 (31.34-59.88)		
	iBM	68.46 ± 23.73 (29.33-108.60)	49.26 ± 13.63 (20.87-63.27)	73.88 ± 22.63 (23.74-107.11)	58.51 ± 19.59 (24.92-84.38)		**p<0.05; OptExp2**
Number of Shots	PA	37.30 ± 13.65 (12.00-61.00)	39.10 ± 15.39 (14.00-70.00)	41.50 ± 12.89 (18.00-58.00)	22.60 ± 8.87 (9.00-42.00)	**p < 0.05**	
	VS	33.90 ± 20.71 (10.00-83.00)	32.20 ± 15.83 (10.00-62.00)	30.20 ± 17.16 (8.00-62.00)	19.80 ± 8.53 (7.00-32.00)	**p < 0.05**	
	pBM	53.70 ± 20.37 (41.00-107.00)	43.30 ± 12.49 (29.00-64.00)	59.40 ± 25.26 (37.00-121.00)	27.40 ± 6.87 (11.00-36.00)	**p < 0.05**	
	iBM	16.84 ± 12.53 (1.00-49.00)	11.97 ± 8.36 (1.00-35.00)	17.35 ± 14.04 (1.00-50.00)	6.00 ± 5.22 (1.00-21.00)	**p < 0.05**	

OptExp1 and OptExp2 are from two experienced planners and OptNov is from one novice planner.

PA, pituitary adenoma; VS, vestibular schwannoma; pBM, post-operative brain metastases; iBM, intact brain metastases; GI, gradient index; PCI, Paddick Conformity Index; RxPercentage, prescription percentage; sBOT, scaled beam-on time.

The bold values mean the difference is significant with p < 0.05.

**Table 2:** Summary of the dosimetric metrics among the inversely optimized and clinical plans for all sites.

Metrics	Site	OptExp1	OptExp2	OptNov	Clinical	p-value Student’s t-test	ANOVA
LON_D0.03cc (Gy)	PA	4.88 ± 2.17 (1.40-7.90)	5.28 ± 2.61 (1.40-9.00)	5.38 ± 2.49 (1.70-8.90)	5.39 ± 2.17 (1.40-8.10)		
RON_D0.03cc (Gy)	PA	4.86 ± 2.09 (2.30-8.70)	5.00 ± 2.46 (2.30-9.80)	5.11 ± 2.34 (2.10-9.10)	5.26 ± 2.78 (2.30-9.80)		
BS_D0.03cc (Gy)	PA	8.09 ± 2.86 (4.40-11.40)	9.58 ± 3.66 (5.30-14.10)	9.02 ± 3.20 (5.10-13.30)	9.45 ± 3.41 (4.90-13.10)		
BS_D0.5cc (Gy)	PA	5.97 ± 2.00 (3.50-8.30)	6.50 ± 2.35 (3.30-9.50)	6.35 ± 2.18 (3.40-9.20)	6.48 ± 2.34 (3.20-8.80)		
Chiams_D0.03cc (Gy)	PA	5.15 ± 0.98 (3.80-6.90)	5.17 ± 1.30 (3.50-7.70)	5.20 ± 1.09 (3.40-6.80)	5.51 ± 1.25 (3.70-7.40)		
BS_D0.03cc (Gy)	VS	10.40 ± 3.32 (3.70-14.10)	11.24 ± 3.24 (5.20-14.00)	11.60 ± 3.42 (4.90-14.70)	10.84 ± 3.19 (4.90-14.40)		
BS_D0.5cc (Gy)	VS	7.51 ± 3.18 (1.80-11.30)	7.62 ± 3.09 (2.50-11.10)	7.84 ± 3.20 (2.30-11.70)	7.65 ± 3.10 (2.30-11.20)		
Cochlea_D0.03cc (Gy)	VS	5.64 ± 2.71 (2.30-11.20)	5.56 ± 2.61 (1.90-10.40)	5.30 ± 1.87 (2.20-8.10)	5.52 ± 2.87 (2.50-12.60)		
Brain_V12Gy (cc)	pBM	38.42 ± 15.34 (11.51-65.28)	40.11 ± 16.99 (12.14-74.12)	37.98 ± 15.94 (11.19-67.02)	43.53 ± 19.86 (17.42-89.79)		
PTV_D 98% (Gy)	pBM	17.04 ± 1.44 (15.30-18.70)	17.13 ± 1.41 (15.40-18.70)	17.20 ± 1.39 (15.50-18.80)	17.06 ± 1.89 (13.90-20.00)		
Cavity_D 98% (Gy)	pBM	19.39 ± 2.19 (15.90-22.10)	19.48 ± 2.16 (16.00-22.20)	19.80 ± 2.31 (16.50-22.40)	18.97 ± 2.11 (15.50-21.60)		
Brain_V12Gy (cc)	iBM	16.88 ± 10.02 (0.45-36.06)	18.86 ± 11.44 (0.57-41.06)	16.98 ± 10.10 (0.58-35.21)	22.16 ± 13.20 (0.54-46.61)	**p < 0.05**	
PTV_D 98% (Gy)	iBM	20.28 ± 2.07 (13.40-25.70)	20.51 ± 1.92 (14.10-23.00)	20.97 ± 2.05 (13.70-24.50)	21.16 ± 2.22 (13.50-24.70)	**p < 0.05**	

OptExp1 and OptExp2 are from two experienced planners and OptNov is from one novice planner.

PA, pituitary adenoma; VS, vestibular schwannoma; pBM, post-operative brain metastases; iBM, intact brain metastases; L/RON, left/right optic nerve; BS, brainstem.

The bold values mean the difference is significant with p < 0.05.

For PA cases, the GI and sBOT were both significantly improved in the inversely optimized plans at the expense of larger number of shots utilized per plan compared to the corresponding clinical plans. No significant differences were observed for any other metrics between the inversely optimized and clinical plans. The results of the ANOVA test indicated that there were no differences in the metrics among the inversely optimized plans by the three planners, except that GIs in the inversely optimized plans created by the novice planner were significantly lower than those in the inversely optimized plans by the two experienced planners.

For VS cases, the low dose spillages were better controlled with significantly lower GIs in the inversely optimized plans compared to the clinical plans, whereas the numbers of shots used per plan were also significantly higher in the inversely optimized plans. No other statistically significant differences in the other metrics were observed between the inversely optimized and clinical plans. Furthermore, all three inversely optimized plans had similar metric values with the only significant differences observed in the PCIs.

For pBM cases, the inversely optimized plans presented better plan qualities with significantly higher PCI and lower GI, but more shots per plan compared to the clinical plans, and there were no significant differences observed among the three inversely optimized plans. For each PTV treated in the iBM cases, despite all PTVs covered with at least 99% of prescription dose, there were significant differences in D_98%_ of PTV, PCI, and GI between the clinical and inversely optimized plans. The volumes of PTV in iBM cases range from 0.02 to 17.62 cc, with a mean of 2.19 cc and a standard deviation of 3.25 cc. While the inversely optimized plans had higher conformality and tighter low dose spillage, which also resulted in lower V_12Gy_ of normal brain tissue, the clinical plans delivered higher radiation dose to the PTVs with higher D_98%_. These differences could probably be explained by the margin deliberately created between the prescription isodose line and PTVs during the manual planning process, especially for small brain metastases. The inversely optimized plans again presented similar plan qualities, except for that the sBOTs in the OptExp2 plans were significantly shorter than those in the OptExp1 and OptNov plans, and that the PTVs in the OptNov plans were prescribed to a significantly lower percentage isodose line.

### BOT Comparison

**Figure 3:**
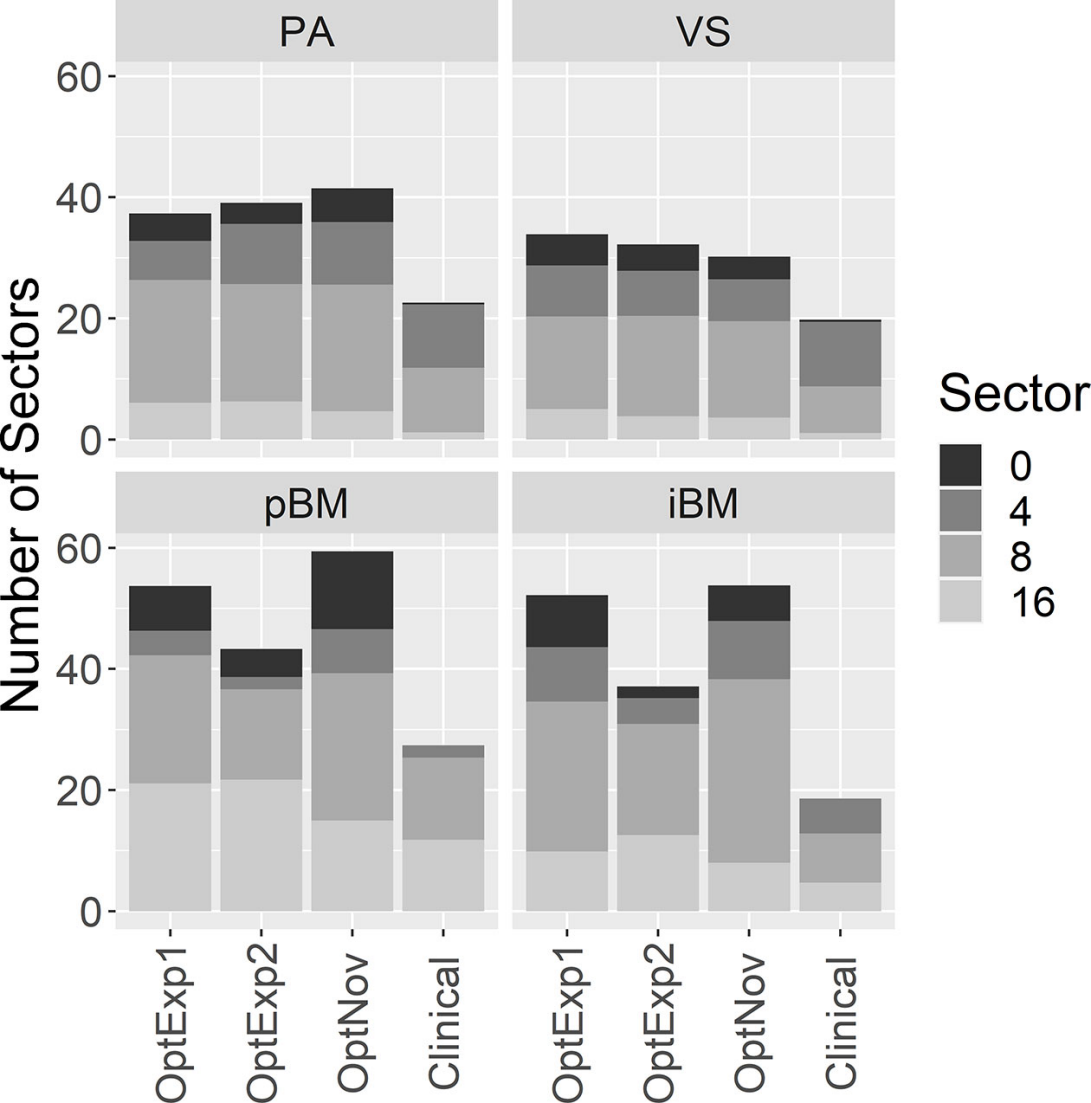
Comparison of the average number of shots and the distribution of different sectors used per plan. PA, pituitary adenoma; VS, vestibular schwannoma; pBM, post-operative brain metastases; iBM, intact brain metastases.

**Figure 4:**
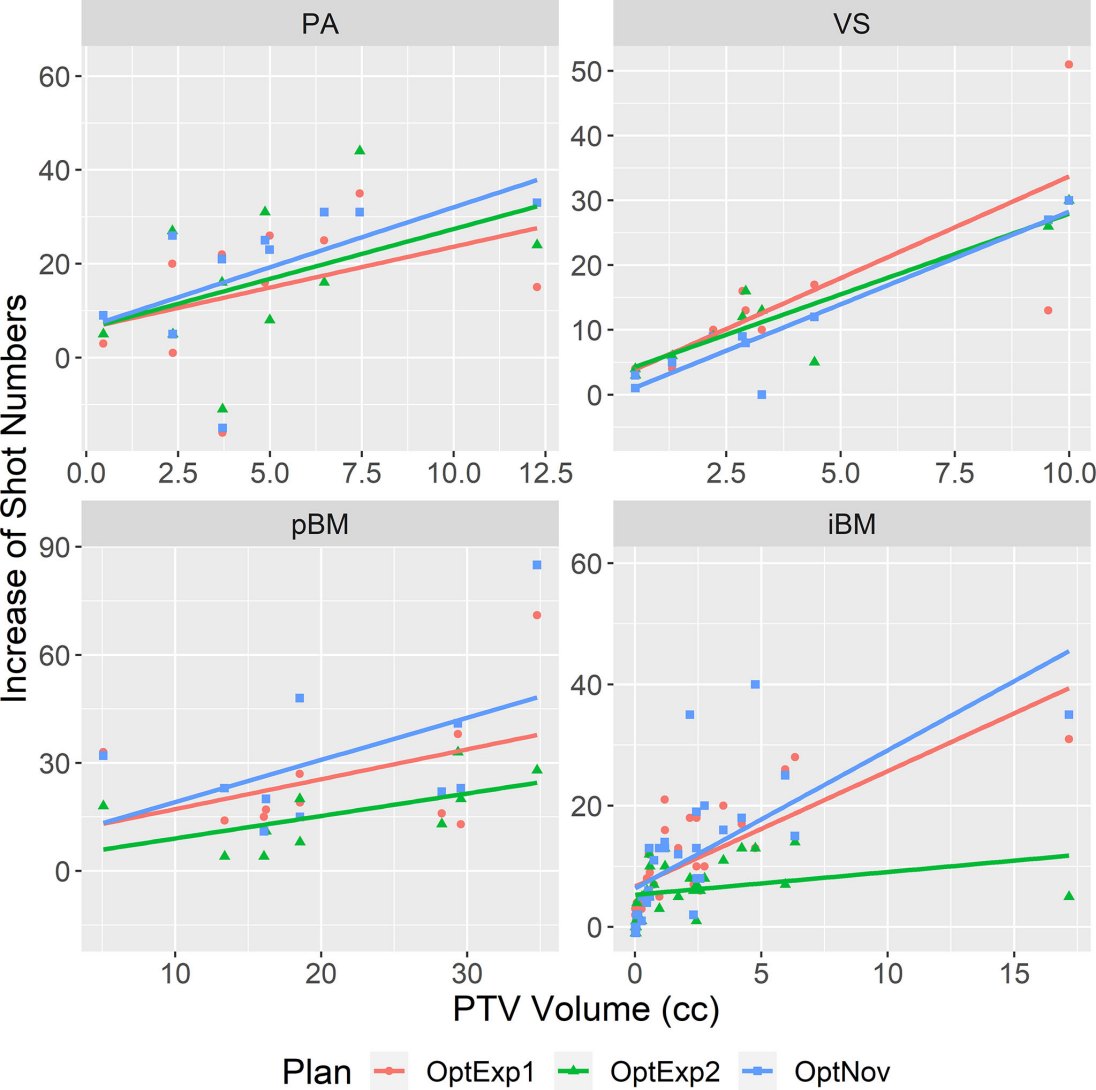
Correlations between the increase of number of shots in the inversely optimized plans and the PTV volume. The linear regression for each site was shown in the straight line. PA, pituitary adenoma; VS, vestibular schwannoma; pBM, post-operative brain metastases; iBM, intact brain metastases.

**Figure 3** summarizes the average numbers of shots and compositions of sectors in the inversely optimized and clinical plans for the four disease sites included in the study. As shown previously, the number of shots used per plan was significantly higher in the inversely optimized plans regardless of treatment sites. Furthermore, the number of shots used per target in the inversely optimized plans positively correlated with the target volume, as illustrated in **Figure 4**. We should also note that significantly more blocked sectors were used in the inversely optimized plans (Chi-squared test; p<0.001) than clinical plans, since planners barely used blocked sectors during the manual planning process in order to avoid the lengthening of sBOTs as a consequence of reduced effective dose rate. The reduction of effective dose rate due to the usage of blocked sector in the inversely optimized plans was compensated by more utilized shots, therefore the sBOTs in the inversely optimized plans remained no worse than those in the clinical plans.

## Discussion

The results of the study revealed that inversely optimized plans generated using the new inverse planning technology in GK Lightning TPS presented comparable quality to clinically approved plans. No consistent differences between the inversely optimized plans generated by experienced and inexperienced users were observed, which implied the persistent performance of the inverse planning technology in GK Lightning TPS which was independent of planner’s experiences. Hence, the implementation of the inverse planning technology may potentially help flatten the learning curve for inexperienced users. To date, the research on the clinical applications of the inverse planning technology in GK is still limited. Wieczorek et al. (11) compared inverse plans optimized by GK Lightning with manual forward plans on 115 lesions and demonstrated that the inverse plans were comparable or superior to forward plans with regard to plan quality metrics. Spaniol et al. (12) performed similar analyses on 38 patients’ plans, as well as evaluated inter-operator variability on one plan for every pathology type. They also showed improved plan quality with GK Lightning, with minimal variability on the operator’s experience. Compared with previous studies, our study not only classified plan quality comparisons into different diagnoses with a wider range of treatment sites, but also investigated planner dependence for all forty plans. Our study provides an independent evaluation of the inverse planning technology and a comparison of GK plans created by experienced and inexperienced users with various disease sites clinically treated with GK.

Overall, the plan quality of inversely optimized plans generated with the inverse planning technology was at least equivalent to the plan quality of those manually created by experienced planners across all disease types, especially the PCI of the inversely optimized plans was significantly better in the pBM and iBM cases. This is primarily due to the nature of malignancy type of lesions and the large separation between the targets and critical organs, so that large shots were often used in forward planning for multiple brain metastases cases to reduce overall treatment time.

The traditional manual forward planning for GK is usually complex and nonintuitive, mostly due to the high degrees-of-freedom of a GK plan. Because of the high dimension of the search space (8), the plan quality of a manually created GK plan largely depends on the experience of the planner, and therefore it can be challenging for an inexperienced planner to create an acceptable GK plan. Even for plans created by experienced planners, it is very likely that these plans are not the optimal solution, especially when the target is large and complex with adjacent OARs present. It was not until the release of Leksel GammaPlan V10.0 (2010) that resolving this limitation of manual forward planning became possible with an inverse planning tool (16). This commercially available tool provides a solution with a two-step optimization process that first identifying the number and location of shots then adjusting the weighting of each shot to achieve the desired plan quality. However, the implementation of this first version of the inverse planning technology has been limited with several drawbacks. The inverse planning tool is unable to create a plan equivalent to a manual plan created by an expert planner unless the targets are regular-shaped with no adjacent OARs. Additionally, it is only suitable for the optimization of one target at a time and it doesn’t account for the existing dose spillage from nearby targets during the optimization.

The optimization technology in GK Lightning has been substantially improved from the previous version. The simultaneous optimization of multiple targets is allowed in GK Lightning and is integrated as a single-step procedure where the placement of shots and the pre-selection of sectors are no longer needed. Furthermore, prescription doses to targets and maximum doses to OARs can be specified prior to optimization, which are especially beneficial for the planning of benign cases presented with adjacent OARs. Additionally, planners only need to manipulate optimization with respect to two optimization objectives: low dose spillage and BOT. This has been simplified compared to prior versions where planners needed to specify coverage, selectivity, GI, and BOT.

We should note that the isodose line prescription percentage is implicitly determined during the optimization. Whereas the lower bound of the prescription percentage of a target is directly correlated with the maximum dose of the target, which can also be specified in the optimization, the upper bound of the prescription percentage can only be adjusted indirectly by increasing the penalty on low dose spillage. This lack of direct manipulation of the prescription percentage imposes challenges to control the dose homogeneity of a target. It has been argued that the internal hotspot created by prescribing to a lower percentage would increase the response of the central hypoxic region of the tumor and therefore result in a higher local control (17), whereas it was also reported that prescribing to a percentage of 70% or higher would not affect local control (18). Despite the lack of consensus, the optimum prescription percentage of 50% is usually preferred at our institution in order to take the advantage of the steepest dose fall-off, with up to 70% isodose line prescriptions allowed in certain cases to increase target conformity; however, when using GK Lightning for small intact brain metastases, we have noticed that the prescription isodose line could be increased to higher than 80% because of the preset penalties on lower target conformity and longer BOT. Therefore, for small intact brain metastases cases, it might be necessary to manually adjust prescription percentage or re-optimize by assigning a higher weighting to low dose spillage and lower weighting to BOT to achieve satisfactory treatment plans.

It is worth noting that the actual delivery time is the sum of the BOT and transition times between consecutive shots. The more shots used in a GK plan, the longer transition time will be added to the total delivery time. In general, inversely optimized plans have a higher number of shots, since the algorithm in the GK Lightning tends to deploy multiple shots of different sector combinations at a single location (10). Thus, the actual delivery time of an inversely optimized plan will be longer than that of a manual forward plan with similar plan quality and BOT. On average, adding one more shot in a GK plan will add an approximately 5-second transition time. Therefore, an inversely optimized plan with 30 more shots would result in additional two and a half minutes in the actual delivery time. Although the impacts are expected to be small for patients immobilized with frame fixation, this increase in the actual delivery time may affect patients treated with frameless fixation in several aspects, including higher likelihood of treatment interruptions due to the intrafraction motion and patient discomfort.

Nevertheless, the inverse planning in GK Lightning improve the workflow of GK treatment. Experienced users may find the automatic planning process advantageous to free themselves from the tedious and lengthy manual planning while focusing on other aspects that need their clinical knowledge and judgments. The overall good quality of inversely optimized plans is beneficial especially for inexperienced users, in that inexperienced users could create plans of comparable quality to those created by experienced users with similar planning time. It should be noted that manual contouring is needed for the inverse planning, thereby additional contouring time is required and should be considered for clinics where the manual forward planning is conducted without contouring.

One limitation of the study is that there was no direct comparison of the treatment planning time between the inversely optimized and clinical plans, since the study was retrospective and treatment planning times were not recorded for manually generated clinical plans. Although treatment planning time of a GK plan, regardless of whether the plan is created manually or with inverse planning, increases for more complex plans, it usually takes substantially more time to manually create plans for multiple large and irregular targets. While the planning time of a manual plan varies from 5 minutes for a single, regular brain metastasis to more than half an hour for a large post-operative cavity, the optimizing of a plan with the inverse planning usually takes less than 15 minutes, despite several iterations of re-optimization. It can be inferred from clinical experiences that the implementation of the inverse planning technology in GK Lighting will help reduce treatment planning time and improve planning efficiency.

## Conclusion

The dosimetric quality of the plans inversely optimized with GK Lightning TPS are comparable to those forwardly planned by an experienced user. The performance of the inverse optimization is user independent, making the inverse planning technology in GK Lightning TPS a promising tool to enable efficient clinical workflow.

## Data Availability Statement

The original contributions presented in the study are included in the article/supplementary material. Further inquiries can be directed to the corresponding author.

## Ethics Statement

The studies involving human participants were reviewed and approved by Rutgers University Institutional Review Board. Written informed consent for participation was not required for this study in accordance with the national legislation and the institutional requirements.

## Author Contributions

XW, TC, and KN led the conception and design of the study. XW, TC, JZ, KN, SD, JW, AC, and NO contributed to acquisition of data. TC, XW, and KN contributed to analysis and interpretation of data. TC, XW, KN, SD, JW, AC, and NY drafted and revised the article. All authors contributed to the article and approved the submitted version.

## Conflict of Interest

The authors declare that the research was conducted in the absence of any commercial or financial relationships that could be construed as a potential conflict of interest.

## Publisher’s Note

All claims expressed in this article are solely those of the authors and do not necessarily represent those of their affiliated organizations, or those of the publisher, the editors and the reviewers. Any product that may be evaluated in this article, or claim that may be made by its manufacturer, is not guaranteed or endorsed by the publisher.
